# Thermographic assessment of upper body muscles in climbers as a methodology for comparing different skill levels

**DOI:** 10.3389/fspor.2025.1662684

**Published:** 2025-11-14

**Authors:** Cristina Comeras-Chueca, Noel Marcen-Cinca, Carlos Valero-Campo, César Berzosa, Eduardo Piedrafita, Héctor Gutiérrez, Pablo Jesus Bascuas, Ana Vanessa Bataller-Cervero

**Affiliations:** 1 Universidad San Jorge, Villanueva de Gállego, Zaragoza, Spain; 2 ValorA Research Group, Health Sciences Faculty, Universidad San Jorge, Zaragoza, Spain

**Keywords:** infrared thermography, upper limbs, muscle thermoregulation, climbing performance, thermoregulatory efficiency

## Abstract

**Background:**

Infrared thermography (IRT) is increasingly used in sports science to monitor muscle temperature changes in athletes. This study aimed to analyze muscle groups of upper body regions warming in climbers, comparing high (HL) and very high-level (VHL) climbers after completing a maximum-difficulty route.

**Methods:**

HL and VHL climbers performed their most challenging route, with thermographic measurements of muscle groups of upper body regions (biceps, elbow flexors, external and internal regions of the forearm, front shoulder, and triceps) taken before, immediately after, and 15 min post-climbing using IRT.

**Results:**

Climbing-specific muscles, such as the biceps, elbow flexors, and forearm regions, warmed significantly immediately after and after climbing. In contrast, secondary muscles regions, like the shoulder and triceps, exhibited minimal changes, indicating their supportive role. Non-specific muscle regions, such as the chest, back, and lower back, exhibited a cooling-rewarming cycle, highlighting their stabilizing role during climbing and contrasting with the greater heat generation of climbing-specific muscle regions. Advanced climbers showed less warming in specific areas—left biceps, right elbow flexor, and forearm—suggesting a more efficient thermoregulatory or metabolic response. This may indicate adaptations associated with advanced climbing performance. An optimized methodology for thermographic studies in climbing should include measurements immediately post-climbing and 15 min later to capture the immediate and ongoing muscle response.

**Conclusions:**

These findings suggest that specific muscles involved in climbing sustain significant temperature increases, with advanced climbers potentially benefiting from enhanced efficiency in muscle thermoregulation, which may contribute to improved performance.

## 1 Introduction

Infrared thermography (IRT) has gained a substantial recognition in sports science as a non-invasive method for assessing skin temperature variations that infer underlying physiological responses during or after sports practice (1). The skin plays a fundamental role in regulating body temperature by acting as an intermediary in thermoregulation and heat transfer processes (2). Through mechanisms such as sweating, vasodilation, and vasoconstriction, the skin helps maintain a stable internal temperature. IRT provides detailed images of heat distribution on the skin, facilitating the analysis of how heat is transferred between superficial and deeper tissues. Through IRT, real-time measurements of surface temperature can be obtained, providing insights into blood flow, metabolic activity, thermoregulation, muscle activation and even fatigue (1, 3). Recent advancements in infrared technology have enabled a higher precision and broader application of IRT, particularly in contexts where acute physiological responses are monitored (4, 5). Athletes exposed to high physical and physiological stress may benefit from infrared skin temperature measurement technology, as this temperature monitoring could be used to optimize performance, assess recovery, detect muscle imbalances, and even prevent injuries (6, 7).

In sports demanding significant strength and endurance, such as climbing, the ability to monitor specific muscle responses through thermal imaging allows for an in-depth understanding of how the body adapts and reacts to dynamic contractions, especially in terms of activation, recruitment and thermoregulation (8), as well as fatigue (9). Previous evidence has also studied that the dynamics of thermal response during and after sports practice reveal valuable information about muscle activation and blood flow across different muscle groups and regions in response to various exercise intensities and types (1, 10). One of the critical areas of investigation within IRT studies is the differentiation in thermal responses based on type of exercise, intensity and athlete level. High-level athletes often demonstrate enhanced thermal efficiency and faster recovery times than amateurs (10, 11) and, on the other hand, temperature is also directly related to workload and execution speed (10, 12).

Climbing is a sport that combines strength, endurance, technique, and mental resilience. In climbing, upper limb muscle strength is key to stabilize and propel the body upwards (13, 14). The skill levels of climbers can be classified by The F-RSD (French Rating Scale of Difficulty) from beginner to expert levels according to the International Rock Climbing Research Association (IRCRA: 18–27) (15). Specifically, these main performance factors involve an improved intermittent isometric resistance to muscle fatigue, along with increased muscle strength and grip force generated mainly by the hand and finger muscles (13, 16). As a result, elite climbers often exhibit distinct physiological responses that reflect a complex interplay between training adaptations, muscle efficiency, and skill-specific demands. Higher level climbers display greater grip strength (17, 18), higher rate of force development, and maximal strength in isometric pull-up (19). Specifically, the key muscle regions most involved during climbing are the biceps, elbow flexors, external and internal regions of the forearm, anterior shoulder, and triceps (20, 21). Additionally, accessory muscle regions such as the chest, costal muscles, trapezius, dorsal muscles, shoulders, and lower back play a supportive role, contributing to overall stability, body control, and force transfer during climbing movements (20, 21). Together, these primary and accessory muscle regions are critical for meeting the complex demands of climbing, enabling both dynamic and sustained efforts required for optimal performance. However, while muscle heating and recovery have been well-documented in other sports, there is a lack of understanding of these processes in climbing.

Temperature increases in specific areas during maximum-effort routes, as measured by IRT, could serve as indicators of muscle strain and activation levels. Despite its growing applications in sports science, IRT research focused on its methodological refinement and reliability across diverse sports contexts or modalities such as climbing remain scarce. For instance, while IRT has demonstrated high specificity (up to 95%) in detecting localized physiological changes (7, 11), there is a lack of consensus regarding measurement methodologies and protocols for different sports modalities, such as climbing, when using IRT to measure skin temperature. This study aimed to address this gap by analyzing the thermal response patterns in the key regions of the upper body muscles of climbers using IRT before, immediately after, and 15 min post-completion of the climbers' most challenging route. Another aim of this study was to compare the skin temperature variations by climbers' level. Such an analysis can provide insights into the dynamics of thermal response in climbing, with special attention to differences based on level. Additionally, this study contributes to refining the methodology for using IRT in climbing, offering practical insights for both researchers and practitioners.

## 2 Materials and methods

### 2.1 Study design

This study utilized a cross-sectional observational design, assessing thermographic responses in climbers of different skill levels, and was conducted in accordance with the ethical guidelines of the Helsinki Declaration of 1964 (revised in Fortaleza, 2013) (22). The study was reviewed and approved by the regional Investigation Ethics Committee (Ethics Committee for Research of the Autonomous Community of Aragón, code assigned: PI13/00100.). It was approved on July 31, 2013. All participants were provided informed consent, which included detailed information about the study procedures and measurements. Participants were explained that participation was voluntary, and participants were informed that they could withdraw at any time without having to provide a reason and without any negative consequences. Participants were also informed that all results would be treated anonymously to ensure confidentiality and obtain objective findings to advance scientific knowledge.

### 2.2 Subjects

The study included 26 experienced male climbers whose skill levels ranged from 6c to 9a on the French Rating Scale of Difficulty (F-RSD). Participants were selected based on a self-reported climbing level classification method, as recommended by Draper et al. (23), ensuring an accurate assessment of their skill level. They were recruited among mountain clubs belonging to the regional sports federation of Aragón, in climbing clubs and institutions. As inclusion criteria, climbers must have completed three onsight climbs with a minimum grade of 6c within the past two years, demonstrate at least five years of experience in sport climbing, and be at least 18 years old. Individuals were excluded if they had any musculoskeletal conditions that could potentially limit or alter their climbing performance. This participant selection process aimed to provide a robust representation of high (HL) and very high-level climbers (VHL), enabling a reliable analysis of muscle thermographic responses in this specific athletic population. The HL climbers were classified with grades from 6c to 7a+, while VHL climbers were classified with grades starting at 7b+. All climbers signed informed consent prior to undergoing the tests and evaluations.

The study initially included a total of 26 participants. However, the sample size for certain analyses was reduced to 18 or 19 participants due to missing values in specific measurements. These discrepancies were not caused by participant exclusions but rather by incomplete data for certain variables. Consequently, the results presented in the tables reflect only the available data for each variable.

### 2.3 Experimental protocol

**Figure 1:**
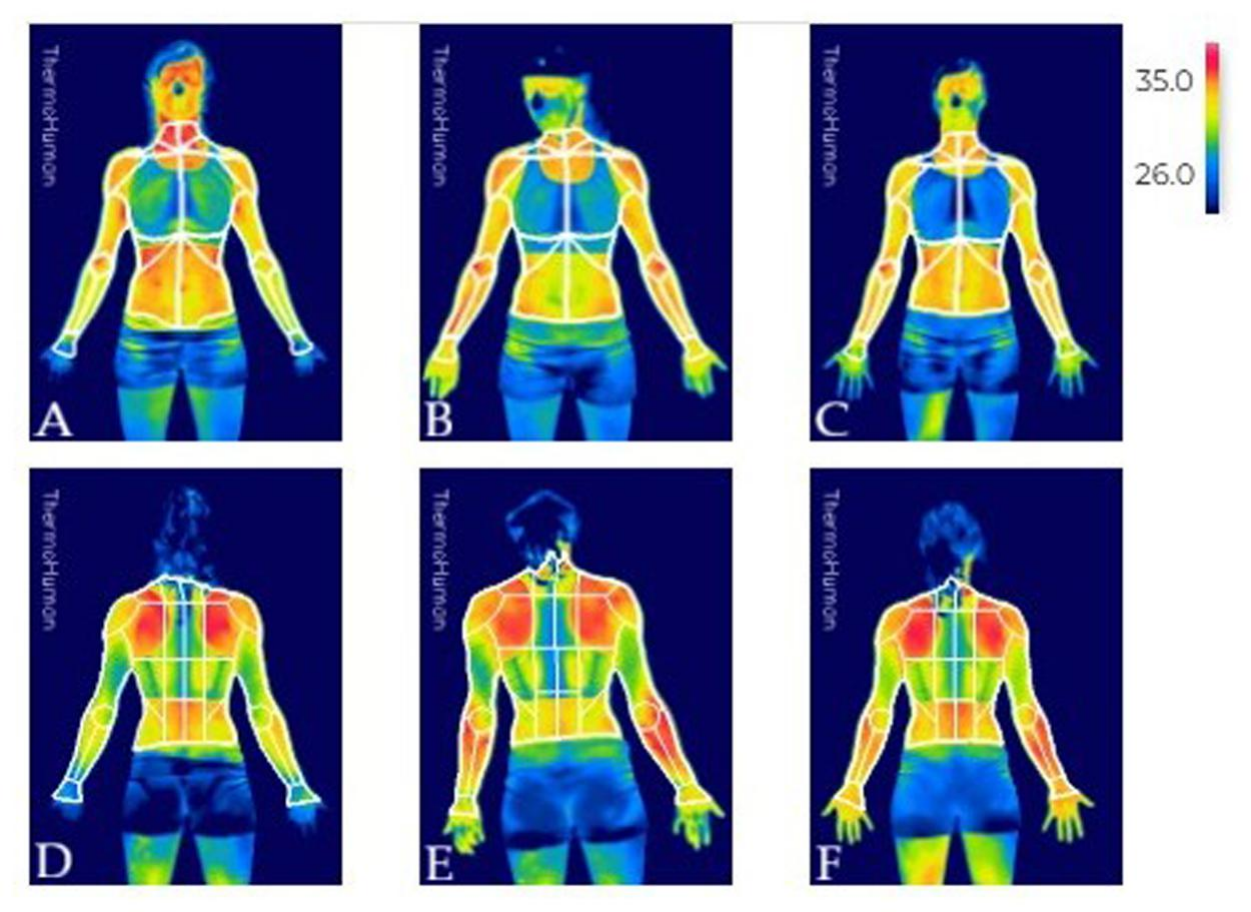
Thermographic images showing the anterior **(A–C)** and posterior **(D–F)** views of the participants at different assessment times. Images **(A,D)** represent the pre-climbing evaluation, **(B)** and **(E)** correspond to the immediate post-climbing assessment, and **(C,F)** show the evaluation 15 min after climbing.

This research was conducted at the climbing gym Dock 39 in Zaragoza, where a dedicated room was equipped to capture thermographic images. All ascents were performed on indoor sport-climbing routes on the same wall sector at Dock 39. Before climbing, participants performed a self-selected warm-up consistent with their usual routine, lasting 15–20 min. To ensure near-maximal exertion, each climber attempted an individualized route of maximum difficulty according to his ability (F-RSD scale), chosen from a preselected and categorized set of routes, and completed it on the first attempt. The climbers ascended the routes as fast as they could. Each participant completed one continuous ascent to top-out. Chalk was allowed and used during the climb. Prior to imaging, visible chalk residue and perspiration were gently removed from the upper-body regions of interest to minimize evaporation-driven cooling in the immediate post-climb measurement. Thermographic images were taken at three critical time points to capture muscle temperature variations: 1) immediately before climbing to establish a baseline; 2) just after completing the route to assess the immediate thermal response to intense exertion; 3) and 15 min post-climbing to monitor short-term recovery patterns regarding possible previous thermographic alterations. An example of these assessments is shown in Figure 1, where the evolution of temperature can be observed over time. The images illustrate how thermal patterns change from baseline through the immediate post-effort state and into the early recovery phase, providing visual insight into the physiological response and subsequent recovery process.

The specific regions of upper body muscles targeted for thermographic imaging included as specific muscle regions the biceps, elbow flexors, external and internal regions of the forearm, front shoulder, and triceps, and as non-specific muscle regions chest, costal, trapezius, dorsal, shoulder and lower back, as these muscle regions are crucial in climbing performance (20, 21). This protocol was designed to yield precise data on temperature fluctuations in primary and supportive climbing muscles.

### 2.4 Thermography

IRT was used to monitor muscle changes in climbers. Images were captured using a FLIR Thermacam E60 infrared camera (FLIR Systems, Wilsonville, OR, USA) featuring an IR detector of 320 × 240 pixels, thermal sensitivity (NETD) <0.05 °C at 30 °C (50 mK), field-of-view 25° × 19°, spatial resolution (IFOV) 1.36 mrad, and spectral range 7.5–13 µm. Thermographic shots were taken immediately before climbing, immediately after climbing, and 15 min post-climb, following guidelines provided by the American Academy of Thermology (24). Thermographic measurements at 15 min post-exercise provide a critical window to assess recovery and physiological responses. Investigations that also evaluate the upper body, such as studies on judokas (25), runners (26) or on endurance athletes (27). These researches support the relevance of using this time point in climbing, where the upper body is heavily engaged, to assess muscle activation, fatigue, and recovery efficiency while enabling standardized comparisons of thermoregulatory adaptations. The mean temperature was used to report the thermography data, as Machado et al. (28) found it provided higher intra-class correlations for both the camera and examiner compared to maximum values and standard deviation, particularly before exercise. Additionally, post-exercise mean skin temperatures showed the most consistency across cameras and evaluators.

All sessions were performed in the same climate-controlled room (21 °C, 60% RH) with doors and windows closed and diffuse ambient lighting (i.e., no drafts and no direct solar radiation). At the start of each session, reflected apparent temperature (RAT) was measured using the crumpled-and-reflattened aluminum foil technique: a small foil patch mounted on cardboard was positioned in the same plane and distance as the participant; with emissivity temporarily set to 1.00, the foil's apparent temperature was recorded and entered as the Reflected Temperature parameter in the camera/software. If RAT differed from room temperature by >1 °C, lighting or participant posture was adjusted and the reading repeated; shiny/metallic objects were removed to minimize reflections. Participants then completed a 10-minute seated acclimation inside the room before baseline imaging. To limit circadian influences, sessions were scheduled within a consistent time-of-day window across participants. Pre-test behaviors were standardized: 12 h abstinence from caffeine and alcohol, 2 h without heavy meals, no creams/lotions/oils on the upper body on the day of testing, and no vigorous exercise for 24 h. The camera was powered on 10 min before each measurement to ensure electronic stabilization. If visible perspiration occurred, the skin was gently blotted and imaging resumed once dry.

**Figure 2:**
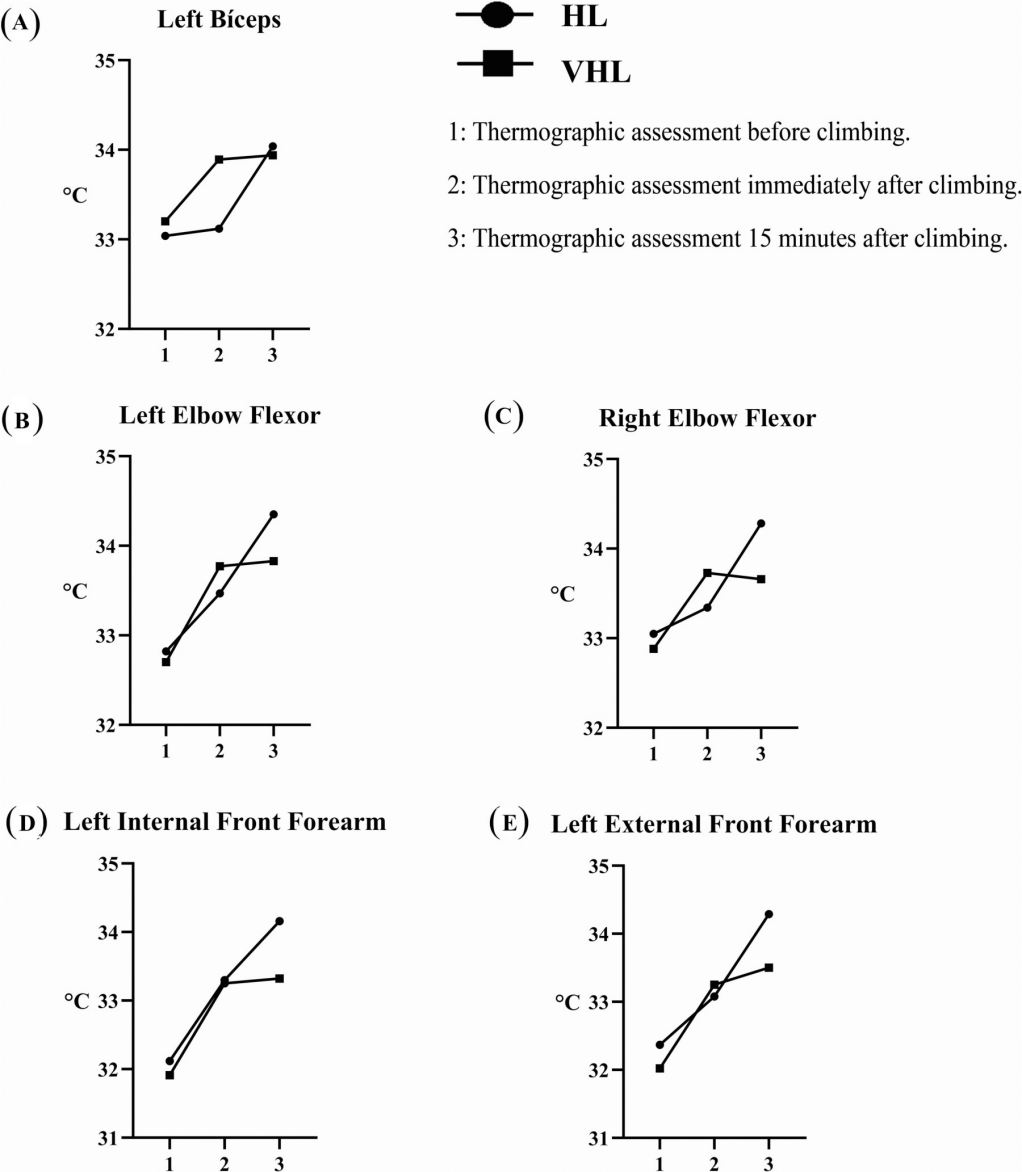
Thermographic evolution of different muscle areas: before (1), immediately after (2), and 15 min after (3) climbing, in two level groups of climbers: HL (circle) and VHL (square). **(A)** left biceps; **(B)** left elbow flexor; **(C)** right elbow flexor; **(D)** left internal front forearm; **(E)** left external front forearm. Results are expressed as thermographic mean value per group (°C) for each assessment time (1–3). *Significant differences (time/level interaction) were observed between (2) and (3) measurements (*p* < 0.05).

Images were taken from anterior and posterior perspectives, ensuring all relevant muscle regions were visible. The camera was positioned 2.5 meters from the participants. The upper body regions targeted for thermography included the front and back shoulder, chest, biceps, costal muscles, elbow flexors, the external and internal front forearm, back trapezius, central dorsal area, dorsal muscles, triceps, lower back, and the external and internal back forearm. The specific body areas analyzed in this study were clearly illustrated in Figure 2. Each region was labeled directly on the diagrams to ensure accurate identification and localization. ROIs were automatically generated in ThermoHuman using predefined, fixed-size templates applied bilaterally and kept constant across pre-, post-0′, and post-15′ images. Anatomical landmarks guided placement and were mirrored left–right. Templates used were: biceps (circular Ø: 30 mm); anterior/posterior shoulder (elliptical 45 mm × 30 mm); elbow flexors (elliptical 40 mm × 25 mm); forearm (volar/dorsal, radial/ulnar; rectangular 50 mm × 25 mm); chest (60 mm × 40 mm); costal (60 mm × 30 mm); upper trapezius (elliptical 45 mm × 30 mm); central dorsal (60 mm × 40 mm); latissimus (50 mm × 30 mm); lower back (bilateral 60 mm × 40 mm).

Temperature readings were analyzed with Thermohuman Software. All thermography measurements took place in a room maintained at a stable temperature of 21 °C with 60% humidity, free from any metallic objects that could interfere with the images. Participants stood without clothes on their upper body on a raised surface avoid direct floor contact, and a skin emissivity value of 0.98 was used for accurate temperature measurement (29).

### 2.5 Statistical analysis

Statistical processing was performed using SPSS version 29.0 for Windows (SPSS Inc., Chicago, IL, USA). Shapiro–Wilk tests were performed to verify the normal distribution of the variables.

To evaluate the effects of climbing on muscle regions of upper body regions warming and compare responses between HL and VHL climbers, a repeated measures analysis of variance (ANOVA) was conducted on thermographic temperature data collected at baseline (pre-climb), immediately post-climb, and 15 min post-climb. Within-subject factors included time and muscle region, while the between-subject factor was climber level (HL vs. VHL). Partial eta-squared (*η*^2^*_p_*) was calculated to assess effect sizes for observed differences, with the commonly accepted cutoff points used to interpret effect size as follows: small effect size, 0.01 ≤ *η*^2^*_p_* < 0.06; medium effect size, 0.06 ≤ *η*^2^*_p_* < 0.14; and large effect size, *η*^2^*_p_* ≥ 0.14 (30). Statistical power (1 − *β*) was computed to ensure sufficient sensitivity of the analysis, with 0.80 considered the reference value for adequate power. *post-hoc* tests were performed where significant main or interaction effects were observed, applying Bonferroni correction. All analyses were conducted with a significance level set at 0.05 (*p* < 0.05), and results were interpreted within the context of observed effect sizes and power calculations.

## 3 Results

Participants had a mean age of 27.3 ± 7.0 years and a mean height of 176.0 ± 7.5 cm. Tables 1, 2 present a detailed analysis of the thermographic measurements for upper body muscle regions across climbers of varying skill levels—categorized as HL and VHL—before, immediately after, and 15 min post-climb. The thermographic measurements targeted the upper limbs, showing that the specific muscle regions involved in climbing were the biceps, elbow flexor, external and internal front forearm, and external and internal back forearm due to their high engagement in grip and pull actions. Front shoulder and triceps were fewer specific muscle regions and displayed a different behavior. The non-specific muscle regions, such as the chest, costal, trapezius, dorsal, shoulder, and lower back, exhibited an even more distinct behavior, with some of them cooling down noticeably by the end of the climb. Because 1 − *β* < 0.80 for several time × level effects, these fndings need to be interpreted cautiously.

**Table 1:** Thermal dynamics of climbing specific muscle regions before, immediately after, and 15 min post-climbing depending on skill level.

Muscle group	Level^1^	n	Pre ± SD (°C)	Post-0′ ± SD (°C)	Post-15′ ± SD (°C)	ANOVA						Pre-climbing vs. immediate post-climbing						Immediate post-climbing vs. 15′ post-climbing					
						Time^2^			Time*level^3^			Time^2^			Time*level^3^			Time^2^			Time*level^3^		
						*p*	*η* ^2^	1 − *β*	*p*	*η* ^2^	1 − *β*	*p*	*η* ^2^	1 − *β*	*p*	*η* ^2^	1 − *β*	*p*	*η* ^2^	1 − *β*	*p*	*η* ^2^	1 − *β*
Right Biceps	1	9	33.24 ± .53	33.26 ± .95	33.89 ± .64	.040	.182	.621*	.778	.468	.046	.380	.050	.140	.420	−.040	.120	.136	.133	.314	.279	.073	.184
	2	9	33.36 ± .58	33.75 ± .98	33.85 ± .69																		
	Total	18	33.30 ± .54	33.50 ± .97	33.87 ± .65																		
Left Biceps	1	9	33.04 ± .60	33.12 ± 1.07	34.04 ± .64	<.001	.465	.997**	.039	.184	.626*	.051	.218	.511	.109	.152	.357	.009	.355	.795*	.019	−.3	.692*
	2	9	33.20 ± .57	33.89 ± .88	33.94 ± .60																		
	Total	18	33.12 ± .57	33.50 ± 1.03	33.99 ± .60																		
Right Elbow Flexor	1	9	33.05 ± .40	33.34 ± 1.02	34.28 ± .81	<.001	.404	.984**	.083	.144	.494	.01	.36	.80**	.16	.12	.28	.082	.177	.414	.049	−.22	.515*
	2	9	32.88 ± .95	33.73 ± 1.11	33.66 ± .67																		
	Total	18	32.96 ± .71	33.53 ± 1.06	33.97 ± .79																		
Left Elbow Flexor	1	9	32.82 ± .50	33.47 ± 1.19	34.35 ± .78	<.001	.61	1**	.12	.124	.428	<.001	.534	.98**	.306	.065	.169	.026	.272	.632*	.053	−.214	.502
	2	9	32.70 ± .86	33.77 ± .93	33.83 ± .63																		
	Total	18	32.76 ± .68	33.62 ± 1.05	34.09 ± .74																		
Right External Front Forearm	1	9	32.21 ± .52	32.91 ± 1.25	34.04 ± .78	<.001	.614	1**	.122	.123	.424	.002	.462	.935**	.603	.017	.079	.008	.363	.809**	.076	−.183	.429
	2	9	32.11 ± .83	33.05 ± .88	33.31 ± .77																		
	Total	18	32.16 ± .67	32.98 ± 1.05	33.67 ± .84																		
Left External Front Forearm	1	9	32.37 ± .52	33.08 ± 1.09	34.29 ± .68	<.001	.74	1**	.037	.186	.633*	<.001	.618	.997**	.193	.104	.249	.001	.496	.961**	.018	−.304	.699*
	2	9	32.02 ± .79	33.25 ± .78	33.50 ± .73																		
	Total	18	32.19 ± .67	33.17 ± .92	33.90 ± .80																		
Right Internal Front Forearm	1	9	32.31 ± .50	33.14 ± .87	33.96 ± .72	<.001	.553	1**	.206	.094	.324	<.001	.549	.985**	.441	.038	.115	.117	.147	.345	.103	−.157	.369
	2	9	32.04 ± .90	33.23 ± .97	33.21 ± .70																		
	Total	18	32.18 ± .72	33.18 ± .90	33.59 ± .79																		
Left Internal Front Forearm	1	9	32.12 ± .66	33.30 ± .91	34.16 ± .73	<.001	.733	1**	.106	.131	.451	<.001	.724	1**	.665	.012	.07	.016	.312	.716*	.036	−.246	.574*
	2	9	31.91 ± .94	33.25 ± .77	33.32 ± .69																		
	Total	18	32.02 ± .80	33.27 ± .82	33.74 ± .82																		
Right External Back Forearm	1	8	32.19 ± .80	33.12 ± .91	33.39 ± 1.48	<.001	.445	.996**	.607	.029	.126	<.001	.578	.995**	.386	.044	.134	.871	.002	.053	.343	−.053	.152
	2	11	31.86 ± .70	33.21 ± .69	33.01 ± .79																		
	Total	19	32.00 ± .74	33.17 ± .77	33.17 ± 1.11																		
Left External Back Forearm	1	8	32.05 ± .83	33.18 ± 1.02	33.38 ± 1.48	<.001	.569	1**	.518	.038	.153	<.001	.652	1**	.32	.058	.162	.927	−.001	.051	.31	−.06	.167
	2	11	31.67 ± .52	33.30 ± .70	33.06 ± .65																		
	Total	19	31.83 ± .68	33.25 ± .82	33.19 ± 1.05																		
Right Internal Back Forearm	1	8	31.66 ± .78	32.69 ± .91	33.25 ± 1.72	<.001	.474	.999**	.399	.053	.2	<.001	.637	.99**	.266	.072	.193	.596	.017	.08	.221	−.087	.224
	2	11	31.28 ± .88	32.86 ± .74	32.64 ± .98																		
	Total	19	31.44 ± .84	32.79 ± .80	32.90 ± 1.33																		
Left Internal Back Forearm	1	8	31.45 ± .78	32.66 ± .91	33.22 ± 1.70	<.001	.6	1**	.242	.08	.294	<.001	.751	1**	.082	.168	.416	.58	.018	.083	.169	−.108	.273
	2	11	31.00 ± .68	33.05 ± .69	32.80 ± .85																		
	Total	19	31.19 ± .74	32.88 ± .79	32.98 ± 1.26																		
	2	11	31.63 ± .56	31.70 ± 1.19	32.15 ± .63																		
	Total	19	31.73 ± .72	31.37 ± 1.22	32.14 ± 1.07																		

Results are expressed as temperature (°C) ± standard deviation. Pre: thermographic assessment before climbing. Post-0′: thermographic assessment immediately after climbing. Post-15′: thermographic assessment 15 min after climbing. SD: standard deviation. ^1^Level: 1 = HL climbers; 2 = VHL climbers. ^2^Time: observed changes regarding the time variable. ^3^Time*level: observed changes for the interaction between time and the level group. *p*: significance level. *η*^2^: eta squared effect size. 1 − *β*: statistical power.

* Effect size with a statistical signification *p* < .05.

** Effect size with a statistical signification *p* < .05 and a statistical power 1 − *β* > .8.

**Table 2:** Thermal dynamics of climbing non-specific muscle regions before, immediately after, and 15 min post-climbing depending on skill level.

Muscle group	Level^1^	n	Pre ± SD (°C)	Post-0′ ± SD (°C)	Post-15′ ± SD (°C)	ANOVA						Pre-climbing vs. immediate post-climbing						Immediate post-climbing vs. 15′ post-climbing					
						Time^2^			Time*level^3^			Time^2^			Time*level^3^			Time^2^			Time*level^3^		
						*p*	*η* ^2^	1 − *β*	*p*	*η* ^2^	1 − *β*	*p*	*η* ^2^	1 − *β*	*p*	*η* ^2^	1 − *β*	*p*	*η* ^2^	1 − *β*	*p*	*η* ^2^	1 − *β*
Right Front Shoulder	1	9	33.73 ± .69	33.25 ± .97	34.18 ± .41	.005	.285	.872**	.367	.058	.182	.22	−.090	.23	.46	.04	.11	<.001	.506	.967**	.139	−.131	.31
	2	9	33.80 ± .57	33.68 ± .93	34.09 ± .57																		
	Total	18	33.77 ± .62	33.47 ± .95	34.14 ± .49																		
Left Front Shoulder	1	9	33.40 ± .62	33.15 ± 1.18	34.21 ± .53	<.001	.356	.958**	.178	.102	.352	.96	.00	.05	.28	.07	.18	.001	.489	.956**	.063	−.2	.467
	2	9	33.44 ± .59	33.72 ± .93	34.06 ± .57																		
	Total	18	33.42 ± .58	33.44 ± 1.07	34.14 ± .54																		
Right Triceps	1	8	32.15 ± 1.14	3.97 ± .99	32.09 ± 1.44	.028	.189	.671*	.091	.131	.478	.079	.17	.421	.056	.199	.491	.006	.364	.837**	.13	−.129	.323
	2	11	31.73 ± .62	31.78 ± 1.28	32.15 ± .83																		
	Total	19	31.91 ± .87	31.44 ± 1.21	32.12 ± 1.09																		
Left Triceps	1	8	31.87 ± .92	3.91 ± 1.18	32.12 ± 1.54	.014	.221	.762*	.151	.105	.384	.157	−.114	.287	.105	.147	.366	.001	.464	.95**	.095	−.155	.386
	2	11	31.63 ± .56	31.70 ± 1.19	32.15 ± .63																		
	Total	19	31.73 ± .72	31.37 ± 1.22	32.14 ± 1.07																		
Right Chest	1	9	33.16 ± 1.34	32.27 ± 1.34	33.31 ± .79	.002	.318	.92**	.972	.002	.054	.02	−.30	.69*	.92	.00	.05	<.001	.584	.993**	.877	−.002	.053
	2	9	33.65 ± .63	32.69 ± 1.04	33.66 ± .72																		
	Total	18	33.41 ± 1.05	32.48 ± 1.18	33.49 ± .76																		
Left Chest	1	9	33.16 ± 1.3	32.26 ± 1.45	33.3 ± .95	<.001	.34	.94**	.94	.00	.06	.01	−.33	.75*	.93	.00	.05	<.001	.586	.994**	.745	−.007	.061
	2	9	33.62 ± .61	32.66 ± .87	33.57 ± .75																		
	Total	18	33.39 ± 1.01	32.46 ± 1.18	33.44 ± .84																		
Right Costal	1	9	32.94 ± 2.13	31.75 ± 1.24	33.22 ± .84	.012	.294	.763*	.738	.011	.071	.026	−.272	.632*	.749	.007	.061	<.001	.724	1**	.268	−.076	.19
	2	9	33.75 ± .57	32.84 ± 1.07	33.87 ± .77																		
	Total	18	33.34 ± 1.57	32.29 ± 1.25	33.54 ± .85																		
Left Costal	1	9	32.97 ± 1.97	31.86 ± 1.33	33.26 ± .81	.007	.322	.82**	.806	.007	.063	.019	−.3	.692*	.961	0	.05	<.001	.738	1**	.42	.041	.122
	2	9	33.71 ± .56	32.64 ± .96	33.73 ± .64																		
	Total	18	33.34 ± 1.46	32.25 ± 1.19	33.5 ± .75																		
Right Back Trapezius	1	9	34.11 ± .63	32.21 ± 1.63	33.31 ± 1.46	<.001	.355	.968**	.142	.109	.396	.001	−.473	.957**	.169	.108	.274	.002	.441	.932**	.587	.018	.082
	2	9	33.43 ± 1.94	32.55 ± 2.13	34.04 ± .6																		
	Total	18	33.72 ± 1.54	32.4 ± 1.89	33.73 ± 1.08																		
Left Back Trapezius	1	9	34.04 ± .75	32.31 ± 1.59	33.28 ± 1.42	.001	.321	.939**	.126	.115	.418	<.001	−.488	.966**	.1	.151	.375	.002	.445	.935**	.627	.014	.075
	2	9	33.42 ± 2.12	32.73 ± 1.86	34.00 ± .7																		
	Total	18	33.68 ± 1.68	32.55 ± 1.72	33.7 ± 1.09																		
Right Dorsal	1	9	33.2 ± .95	32.03 ± 1.84	33.08 ± 1.6	.001	.329	.947**	.603	.029	.127	.006	−.365	.837**	.376	.046	.138	<.001	.49	.967**	.63	−.014	.075
	2	9	33.31 ± .54	32.67 ± .97	33.5 ± .73																		
	Total	18	33.27 ± .72	32.4 ± 1.39	33.32 ± 1.15																		
Left Dorsal	1	9	32.97 ± .94	31.96 ± 1.93	33.08 ± 1.6	.002	.309	.926**	.689	.022	.106	.014	−.305	.732*	.466	.032	.109	<.001	.511	.978**	.498	−.027	.1
	2	9	33.32 ± .56	32.74 ± 1.00	33.5 ± .73																		
	Total	18	33.17 ± .74	32.41 ± 1.47	33.32 ± 1.15																		
Right Back Shoulder	1	9	33.46 ± .86	32.22 ± 1.71	33.35 ± 1.52	.001	.318	.936**	.446	.046	.18	.011	−.321	.762*	.281	.068	.183	.001	.48	.961**	.566	−.02	.086
	2	9	33.36 ± .49	32.82 ± 1.13	33.66 ± .64																		
	Total	18	33.4 ± .65	32.57 ± 1.39	33.53 ± 1.07																		
Left Back Shoulder	1	9	33.2 ± .92	32.09 ± 1.81	33.37 ± 1.67	.002	.315	.933**	.32	.065	.241	.027	−.257	.629*	.212	.09	.232	<.001	.523	.982**	.239	−.08	.21
	2	9	33.28 ± .47	32.94 ± .96	33.66 ± .64																		
	Total	18	33.25 ± .68	32.58 ± 1.4	33.54 ± 1.15																		
Right Lower Back	1	9	32.58 ± 1.16	31.8 ± 2.14	32.16 ± 1.81	<.001	.441	.996**	.895	.007	.066	<.001	−.509	.977**	.692	.009	.067	<.001	.562	.993**	.752	−.006	.061
	2	9	33.08 ± .69	31.61 ± 1.25	32.8 ± .88																		
	Total	18	32.86 ± .92	31.27 ± 1.68	32.53 ± 1.34																		
Left Lower Back	1	9	32.5 ± 1.09	31 ± 1.96	32.1 ± 1.9	<.001	.452	.997**	.985	.001	.052	<.001	−.559	.992**	.878	−.001	.052	<.001	.537	.987**	.892	.001	.052
	2	9	33.17 ± .72	31.58 ± 1.33	32.75 ± .88																		
	Total	18	32.89 ± .93	31.34 ± 1.6	32.47 ± 1.39																		

Results are expressed as temperature (°C) ± standard deviation. Pre: thermographic assessment before climbing. Post-0′: thermographic assessment immediately after climbing. Post-15′: thermographic assessment 15 min after climbing. SD: standard deviation. ^1^Level: 1 = HL climbers; 2 = VHL climbers. ^2^Time: observed changes regarding the time variable. ^3^Time*level: observed changes for the interaction between time and the level group. *p*, significance level; *η*^2^, eta squared effect size, 1 − *β*, statistical power.

* Effect size with a statistical signification *p* < .05.

** Effect size with a statistical signification *p* < .05 and a statistical power 1 − *β* > .8.

It is important to note that while the tables present data separately for right and left sides, the analysis incorporates the mean differences between these sides across the different temporal measurements. This calculation provides a more comprehensive view of the overall thermal dynamics by synthesizing the behavior of both sides, which may help highlight broader trends and patterns that might otherwise be obscured by focusing solely on individual side-specific data. By doing so, the analysis avoids unnecessary redundancy and allows for a more streamlined interpretation of the results, while maintaining the detailed right and left side values available in Tables 1, 2.

### 3.1 Specific muscle regions

The muscle regions that appear to be most engaged in climbing are the elbow flexor and the external and internal front forearm, which seem to follow a common pattern of warming immediately after climbing, with some of them continuing to increase in temperature 15 min post-climbing. The left biceps follows this trend observed in these specific muscle regions, showing a temperature increase after climbing, although no statistical significance (*p* = 0.051). These muscle regions did not show differences in temperature increase immediately post-climbing by skill level; however, differences emerged 15 min after, with greater efficiency observed in VHL climbers.

#### 3.1.1 Pre-climbing vs. immediate post-climbing

Temperature measurements recorded immediately post-climbing showed significant increases in all specific muscle regions compared to the pre-climbing baseline in both HL and VHL climbers. Temperature increases were statistically significant for the right and left elbow flexor (*p* = 0.01 and *p* < 0.001, respectively), the right and left external front forearm (*p* = 0.002 and *p* < 0.001, respectively), the right and left internal front forearm (*p* < 0.001 for both), the right and left external back forearm (*p* < 0.001 for both), and the right and left internal back forearm (*p* < 0.001 for both).

Regarding the temperature increases across measured muscle regions, the elbow flexor rose by 0.3 ± 1.0 °C and 0.7 ± 0.9 °C, the external front forearm increased by 0.6 ± 1.0 °C and 0.9 ± 1.0 °C, the internal front forearm increased by 0.9 ± 0.8 °C and 1.1 ± 1.0 °C, the external back forearm increased by 0.9 ± 1.4 °C and 1.5 ± 0.5 °C, and the internal back forearm increased by 1.1 ± 1.3 °C and 1.8 ± 0.5 °C for HL and VHL climbers, respectively.

The repeated measures ANOVA revealed a significant effect of time on temperature increases across muscle regions; however, no significant interaction was found between time and climbers skill level (time*level effect), indicating that temperature changes over time were consistent across both HL and VHL climbers, as can be seen in Table 1.

#### 3.1.2 Immediate post-climbing vs. 15 min after climbing

The analysis of post-climbing thermal dynamics, using repeated measures ANOVA, reveals significant interactions between time and climbers' skill level. Figure 2 illustrates the thermal responses across different muscle regions for both HL and VHL climbers at various assessment times. Specifically, the findings show that HL climbers continue to exhibit temperature increases in the left biceps (0.9 ± 0.6 °C), elbow flexor (0.9 ± 0.5 °C), and external and internal front forearm (1.2 ± 0.6 °C and 0.9 ± 0.5 °C, respectively). In contrast, VHL climbers demonstrate significantly smaller increases in left biceps temperature (0.6 ± 0.8 °C) and in the external and internal front forearm (0.2 ± 0.9 °C and 0.1 ± 0.9 °C, respectively), with the elbow flexor even showing a temperature decrease (−0.6 ± 1.3 °C). This trend may be attributed to the lower relative effort of VHL climbers, reflecting reduced fatigue, lower metabolic byproduct accumulation (e.g., nitric oxide, lactate, CO2), and decreased muscle compression during exercise. However, these results should be interpreted with caution, as statistical power (1 − *β*) was computed to assess the sensitivity of the analysis; however, it was below the threshold of 0.80, which is commonly considered the reference value for adequate power. This limitation suggests that the sample may not have been sufficient to fully capture all potential interactions, and further research with a larger sample size may be warranted to confirm these findings.

### 3.2 Non-specific muscle regions

The thermographic analysis revealed distinct thermal behaviors in non-specific muscle regions, as can be seen in Table 2. Unlike other muscle regions, the triceps and front shoulder maintained their temperature immediately after climbing and displayed an increase within the first 15 min post-climbing. HL climbers experienced a temperature increase of 0.8 ± 0.6 °C in the triceps and 1.0 ± 0.7 °C in the front shoulder, while VHL climbers showed a temperature increase of 0.1 ± 1.0 °C in the triceps and 0.4 ± 0.7 °C in the front shoulder. Although a trend toward improved thermoregulation in HL climbers can be observed, no significant differences were found between groups.

Table 2 details the thermographic behavior of non-specific muscle regions—including the chest, costal muscles, back trapezius, dorsal muscles, back shoulder, and lower back—highlighting a consistent cooling trend immediately after climbing, followed by significant recovery within 15 min post-climbing.

Among these, the lower back exhibited the most pronounced cooling immediately after climbing, with temperature drops of −1.8 ± 1.7 °C. The back trapezius, chest and costal muscle regions also experienced noticeable cooling immediately after climbing, with mean reductions of −1.3 ± 1.5 °C, −1.1 ± 1.4 °C and −1.3 ± 1.7 °C respectively across all participants. Recovery patterns were consistent across groups, with temperatures increasing by 1.3 ± 1.5 °C, 1.0 ± 1.1 °C and 1.2 ± 1.3 °C on average. The dorsal muscles and back shoulder displayed milder cooling trends. The dorsal muscles cooled by −0.9 ± 1.3 °C on average, and recovery for the dorsal reached 0.9 ± 0.9 °C. Similarly, the back shoulder cooled by −0.9 ± 1.4 °C across all climbers, and recovery increases in the back shoulder were 0.9 ± 1.0 °C.

By 15 min post-climbing, as can be seen in Table 2, the chest, costal muscles, back trapezius, dorsal muscles, back shoulder, and lower back muscle regions displayed temperature recovery to values near or slightly above their pre-climbing baselines. Moreover, more experienced (VHL) climbers exhibited a trend of smaller temperature drops and faster recovery within 15 min post-climbing compared to less experienced (HL) climb.

## 4 Discussion

The skin is key to body temperature regulation, managing thermoregulation and heat transfer via sweating, vasodilation, and vasoconstriction. IRT offers detailed heat maps of skin. The results of the present study, which explore muscle regions of upper body thermal responses in climbing, showed significant temperature increases in climbing-specific muscle regions, mainly elbow flexors, and forearm muscle regions, immediately after climbing, with continued increases during the recovery phase. Conversely, secondary muscle regions like the shoulder and triceps exhibited minimal thermal changes, highlighting their stabilizing role. Advanced climbers demonstrated greater thermoregulatory efficiency. These findings align with existing literature on exercise thermoregulation, offering valuable insights into climbing-specific physiological adaptations.

### 4.1 Specific muscle regions

The elbow flexor, external, and internal front forearm muscle regions showed significant warming immediately after climbing, with some continuing to heat up during recovery, while VHL climbers displayed greater thermoregulatory efficiency 15 min post-climb. The prominent importance of the strength, endurance, and power of the forearm muscle regions, and thus specifically grip strength, specifically in the fingers, as a key performance factor in climbers, has been demonstrated in previous scientific evidence (16, 17).

The findings on the variations in temperature across the more specifically involved muscle regions align with previous studies (4, 9, 31, 32). The thermographic behavior observed in the elbow flexors and forearm muscle regions aligns with findings from a study on unilateral training, which indicates that muscles directly engaged in exercise display a marked and sustained increase in skin temperature due to heightened metabolic activity and delayed heat dissipation (31). Additionally, the delayed clearance of heat post-exercise could be influenced by vasoconstriction in non-active areas and increased blood flow to the exercised regions, a phenomenon previously reported in high-demand activities like strength training. In aerobic and strength exercises, skin temperature has been observed to decrease due to vasoconstriction in inactive areas but increase in active muscles due to blood flow and intensified metabolic activity during exercise (32). However, unlike the immediate post-exercise period, at 15 min post-exercise, blood flow redistribution becomes more pronounced, with the primary muscles engaged receiving additional blood flow due to reactive hyperemia. Furthermore, another possibility worth considering in climbing, as a strength-dominant sport involving high levels of muscle compression, is that muscles exhibiting higher temperatures at 15 min post-climb may have experienced reduced blood perfusion due to compression, leading to greater fatigue accumulation. Priego-Quesada et al. (9) also evidenced an increase in skin temperature in the regions overlying active muscles in response to incremental cycling exercise due to the increased blood flow and metabolic activity during exercise. Another study about acute responses to a pyramidal cycling protocol showed that calf temperature was inversely related to heart rate and perceived exertion, while directly associated with exercise duration (4). Results from previous studies found that regions of interest associated with high activation displayed significant warming, emphasizing the localized nature of thermal responses (3). However, climbing introduces variability in the use of limbs and muscle regions due to the unique movement patterns required by routes of differing difficulty. The observed thermal responses in climbers' biceps and forearms in this study may reflect such varied demands.

Notably, our results showed that these climbing-specific muscle regions exhibited not only significant immediate increases but also continued warming during the recovery period (15 min post-climb). These results are in line with Rojas-Valverde et al. (6), who emphasized that endurance-related activities often induce prolonged post-exercise thermoregulatory adjustments. In the context of climbing, this may be attributed to the high metabolic demand and delayed clearance of heat from muscles engaged in prolonged gripping and pulling actions, in addition to being characterized by isometric contractions. However, execution speed and effort duration are variables that can influence skin temperature at the end of the effort and subsequent recovery, as different execution speeds and workloads could determine a rapid and marked difference in skin temperature responses (12). Other variables such as body fat percentage and muscle density can significantly influence thermographic readings by altering heat dissipation and retention patterns, as stated by Neves et al. (33).

One of the main focuses of the article was the comparison between HL and VHL climbers, finding reduced warming in VHL climbers after 15 min post-climbing, particularly in the left biceps and forearm regions. VHL climbers also exhibited a trend, although not statistically significant compared to HL climbers, towards a greater increase in muscle temperature upon completing the climb. One possibility worth considering is that lower-level climbers may experience higher levels of muscular compression due to their relatively lower strength and higher effort rates during the ascent, particularly in forearm muscles. This could lead to a greater accumulation of metabolic byproducts and reactive hyperemia once the compression is relieved, explaining, at least in part, the more pronounced temperature increases observed 15 min post-climb in comparison with VHL climbers. Prior studies on thermographic comparisons between trained and untrained subjects demonstrated that training enhances the ability to rapidly increase localized temperature in response to exercise, with faster temperature elevation observed in the more trained individuals (11). Therefore, our findings align with previous studies (31) that suggest that training level influences skin temperature regulation during exercise, showing that lower-trained participants exhibited a greater increase in absolute skin temperature values compared to higher-trained participants, likely due to the higher relative effort required by the lower-trained individuals. This can be explained by the fact that trained athletes recruit more synergistic muscles, reducing the load on agonists and improving thermoregulation (31). Similarly, Masur et al. (34) noted that thermoregulatory patterns in team and individual sports were influenced by training level and metabolic demands, with higher-level athletes showing more efficient heat dissipation and recovery post-exercise (7). This underscores the potential of IRT to identify performance adaptations and guide training strategies in climbing and other sports.

Although the results of the present study did not show significant differences by skill level immediately post-climb, possibly due to the higher difficulty of the routes completed by the more advanced climbers, they do indicate that, 15 min post-climb, lower-level climbers continue to show increases in temperature in the left biceps, elbow flexor, and both the external and internal front forearm, while more advanced climbers demonstrate significantly smaller increases in left biceps temperature and in the external and internal front forearm, with the elbow flexor even showing a temperature decrease. However, these results must be viewed with caution, as the statistical power (1 − *β*) fell below the 0.80 threshold.

Additionally, previous evidence has also compared climbers of different levels, finding that elite climbers have greater finger strength and arm endurance than advanced climbers (14). Another study by Stien et al. (19) comparing intermediate and advanced climbers with elite climbers found no differences in maximal force and rate of force development between intermediate and advanced climbers, but elite climbers exhibited higher rate of force development and maximal force, so their ability to generate force quickly and with greater intensity likely contributes to their better performance. This superior handgrip and finger strength and endurance, and higher rate of force development might explain the trend of greater temperature increases observed in higher-level climbers and their better thermoregulation, as evidenced by a smaller rise in temperature 15 min after climbing. Skin temperature appears to correlate with electrical manifestations of fatigue (9) and reflect physiological responses, including metabolic activity, blood flow for muscle oxygenation, and thermoregulation (3). Therefore, it would be advisable to include specific training for these muscles in climbers to enhance their performance.

### 4.2 Non-specific muscle regions

In non-specific muscle regions, including the triceps and front shoulder, VHL climbers showed a greater temperature increase, but no statistically significant differences were found between groups. A very recent systematic review highlights the importance of a multidimensional approach to climbing performance, emphasizing secondary muscle regions such as the shoulder, back, or even the core and legs (13), although temperature was not measured in these areas in the present study. Our findings are in accordance with Escamilla-Galindo et al. (31), who noted that non-primary muscles in unilateral training protocols exhibited smaller thermal responses due to their supportive role rather than direct involvement in high-intensity contractions.

The distinct behavior of these secondary muscle regions highlights the specificity of thermographic responses in reflecting the functional role of muscle groups during exercise. This supports the use of IRT as a precise tool for identifying muscle recruitment patterns in climbing, where specific muscles bear the brunt of the workload while others act as stabilizers.

The results also showed that non-specific muscle regions, such as the chest, costal muscles, back trapezius, dorsal muscles, back shoulder, and lower back, experienced a consistent cooling right after climbing, followed by noticeable recovery within 15 min. This cooling-rewarming cycle underscores the supportive, stabilizing role of these muscle groups during climbing, with their thermographic behavior differing from that of climbing-specific muscle regions, which generate more metabolic heat due to greater engagement.

The observed cooling-rewarming pattern in non-specific muscle regions aligns with previous studies highlighting the thermoregulatory role of inactive or stabilizing muscles during exercise. This phenomenon may be attributed to vasoconstriction in these areas, mediated by the action of the sympathetic autonomic nervous system and catecholamines during exercise, as observed during aerobic exercise, where inactive regions exhibit decreased blood flow and temperature, while active muscles experience vasodilation and increased heat production (2, 10, 32, 35, 36). The smaller thermal response observed in secondary muscle regions is consistent with findings in strength training protocols where non-primary muscles exhibit smaller temperature changes due to lower metabolic demands (35). Further supporting this, a systematic review by Masur et al. (34) highlighted the role of IRT in detecting temperature asymmetries and cooling-rewarming cycles in inactive or stabilizing muscles across multiple sports and these insights suggest that such thermographic patterns are consistent across diverse physical activities and could provide valuable benchmarks for climbing-specific muscle function. These stabilizing muscles, despite their supportive function, contribute to performance by facilitating efficient force transfer and reducing the load on specific muscles, as suggested by studies on muscle-specific thermoregulation (2). Merla et al. (10) demonstrated that skin temperature in runners decreased during exercise due to heat dissipation driven by sympathetic vasoconstriction in secondary regions, which optimizes blood flow redistribution towards active muscles. However, skin temperature increased post-exercise in regions with high blood flow, likely due to reactive hyperemia and the elevated metabolic demand of previously active muscles. This pattern, partially mirrored in climbing, underscores the interplay between vasoconstriction and vasodilation in maintaining thermoregulatory balance. Additionally, the increase in post-exercise temperature could reflect delayed heat clearance from muscles subjected to intense contractions, particularly in sports like climbing, where sustained isometric efforts may impede immediate perfusion and amplify recovery demands.

Interestingly, more experienced climbers exhibited a trend of smaller temperature drops and faster recovery within 15 min post-climb compared to less experienced climbers. Although no significant differences were found between the two groups, the pronounced cooling in HL climbers compared to VHL climbers may suggest delayed thermoregulatory efficiency, with the latter group displaying more moderate temperature shifts and faster stabilization. This difference could be attributed to improved vascular and metabolic adaptations in VHL climbers, aiding in more effective heat dissipation and recovery after exertion. Similar trends have been observed in dynamic warm-ups, where faster reductions in skin temperature correlate with enhanced performance metrics, such as in countermovement jumps (36).

Additionally, muscles in regions such as the shoulder and elbow, which may play a more stabilizing role in climbing by controlling shoulder and elbow extension, might experience higher isometric workload and compression during exercise in less experienced climbers. This could impair blood flow and lead to greater temperature increases 15 min post-climb in HL climbers compared to VHL climbers, who likely utilize these muscles more efficiently.

From a training perspective, focusing on strengthening these stabilizing muscles could improve their isometric endurance and vascular efficiency, enhancing both performance and recovery in climbers. Developing their stabilizing role could also contribute to better force transfer and reduced fatigue during climbs, particularly for less experienced climbers.

### 4.3 Limitations, practical applications, and future research

This study, while offering valuable insights, has several limitations that need to be addressed in future research. The relatively small sample size restricts the generalizability of the findings and may have affected statistical power (1 − *β*), which fell below the recommended threshold of 0.80 in some analyses. This limitation could hinder the detection of smaller but meaningful effects. Additionally, the cross-sectional design prevents the evaluation of longitudinal training adaptations, which could offer a more comprehensive understanding of thermoregulatory efficiency and muscle activation over time. Temperature measurements were taken before climbing, immediately after, and 15 min post-climb; however, the absence of real-time monitoring during the climbing activity limited the analysis of dynamic thermal responses. Furthermore, the lack of standardized protocols for infrared thermography (IRT) in climbing-specific contexts poses challenges for consistent data interpretation. The findings of the present study also provide practical implications for optimizing thermographic assessment in climbing. Consistent with recommendations by Moreira et al. (1), our methodology, involving pre-, post-, and 15-minute post-climb measurements, effectively captured both immediate and delayed thermal responses. The controlled environmental conditions and use of standardized IRT protocols ensured reliable data collection​. However, including additional post-climb measurements at 5, 10, 20, and 30 min or even post 24 and 72 h in future studies could provide a more detailed understanding of the recovery phase and refine methodologies to address these limitations (34). However, it is important to note the limited applicability of such long periods, like one- or two-days post-activity, as climbing fatigue is highly localized and acute. Therefore, a standardized protocol that considers factors such as sports modality (duration of effort, type of muscle contraction, environmental conditions, etc.). This need for a standardized protocol is mentioned in a very recent systematic review of methods (6). Additionally, previous studies emphasized the importance of considering body composition and environmental conditions when interpreting IRT data. The integration of such variables into standardized protocols could enhance the accuracy and applicability of findings across sports disciplines (25, 34).

Despite these challenges, the findings of this study demonstrate the applicability and utility of IRT as a non-invasive tool for evaluating climbing-specific thermal responses. The methodology employed in this research, which included pre-, post-, and 15-minute post-climb measurements, effectively captured both immediate and delayed thermal responses, offering a standardized approach that can be applied in future investigations. Additionally, the identification of thermal patterns in specific muscles allows for the development of personalized training regimens aimed at improving thermoregulatory efficiency and climbing-specific muscle endurance. The ability to distinguish thermoregulatory differences between climbers of varying skill levels further highlights IRT's potential as a metric for assessing training adaptations. For example, the greater thermoregulatory efficiency observed in very high-level climbers can serve as a benchmark for guiding the development of less experienced climbers. Moreover, the detection of abnormal temperature variations provides an opportunity for injury prevention by identifying areas at risk of tendinopathies or inflammations before clinical symptoms manifest.

Future research should address the limitations identified in this study and expand upon its findings. Longitudinal studies are needed to explore how thermoregulation and muscle-specific thermal patterns adapt over time with training. Real-time monitoring of thermal responses during climbing could offer dynamic insights into how muscle activation and heat production evolve throughout the activity. It would also be valuable to investigate differences in thermal responses across climbing disciplines, such as bouldering and lead climbing, and routes of varying difficulty. Additionally, the skin regulates body temperature through sweating, vasodilation, and vasoconstriction, so IRT captures heat distribution, helping study heat transfer between surface and deeper tissues. By monitoring skin temperatures in different areas of the body, researchers can identify patterns of vasodilation and vasoconstriction. However, a better understanding of the interaction between this superficial tissue and deeper tissues is needed. Integrating IRT with complementary tools like electromyography or blood lactate monitoring could deepen the understanding of the relationship between muscle activation and thermoregulation (25, 34).

Future investigations should also explore the influence of physiological variables, such as body composition, muscle density, and gender, on heat dissipation and retention patterns. Finally, establishing universal guidelines for the use of IRT in sports, considering environmental conditions, exercise intensity, and muscle contraction types, will help standardize methodologies and enhance the reproducibility of findings.

By addressing these areas, future research can further establish IRT as a robust tool for performance optimization, injury prevention, and a deeper understanding of physiological adaptations in climbing and other demanding sports.

## 5 Conclusions

This study highlights the utility of IRT in evaluating muscle-specific thermal responses in climbing, revealing significant differences in warming patterns between primary and secondary muscles and between climbers of different skill levels. Moreover, the enhanced thermoregulatory efficiency observed in very high-level climbers underscores the potential of IRT to provide insights into training adaptations and performance optimization. By refining methodologies and exploring longitudinal effects, future research can further enhance our understanding of thermoregulation and muscle function in climbing and other high-demand sports.

## Data Availability

The datasets presented in this study can be found in online repositories. The names of the repository/repositories and accession number(s) can be found below: the dataset generated and analyzed during the current study is publicly available in the Zenodo repository and complies with the FAIR principles (Findable, Accessible, Interoperable, and Reusable). The dataset can be accessed at: https://doi.org/10.5281/zenodo.15846696.
